# Quantitative FLAIR MRI in Amyotrophic Lateral Sclerosis

**DOI:** 10.1016/j.acra.2017.04.008

**Published:** 2017-10

**Authors:** Jeremy Fabes, Lucy Matthews, Nicola Filippini, Kevin Talbot, Mark Jenkinson, Martin R. Turner

**Affiliations:** a Centre for Functional Magnetic Resonance Imaging of the Brain, Oxford University, John Radcliffe Hospital, West Wing Level 6, Oxford OX3 9DU, UK; b Nuffield Department of Clinical Neurosciences, Oxford University, John Radcliffe Hospital, West Wing Level 6, Oxford OX3 9DU, UK; c Department of Psychiatry, Oxford University, John Radcliffe Hospital, Oxford, UK; d Oxford University Centre for Magnetic Resonance Research, John Radcliffe Hospital, West Wing Level 6, Oxford OX3 9DU, UK

**Keywords:** Amyotrophic lateral sclerosis, motor neuron disease, biomarker, neuroimaging, phenotype, prognosis

## Abstract

**Rationale and Objectives:**

T2-weighted magnetic resonance imaging (MRI) hyperintensity assessed visually in the corticospinal tract (CST) lacks sensitivity for a diagnosis of amyotrophic lateral sclerosis (ALS). We sought to explore a quantitative approach to fluid-attenuated inversion recovery (FLAIR) MRI intensity across a range of ALS phenotypes.

**Materials and Methods:**

Thirty-three classical ALS patients, 10 with a flail arm presentation, and six with primary lateral sclerosis underwent MRI at 3 Tesla. Comparisons of quantitative FLAIR intensity in the CST and corpus callosum were made between 21 healthy controls and within patient phenotypic subgroups, some of whom were studied longitudinally.

**Results:**

Mean FLAIR intensity was greater in patient groups. The cerebral peduncle intensity provided the strongest subgroup classification. FLAIR intensity increased longitudinally. The rate of change of FLAIR within CST correlated with rate of decline in executive function and ALS functional rating score.

**Conclusions:**

FLAIR MRI encodes quantifiable information of potential diagnostic, stratification, and monitoring value.

## Introduction

The neurodegenerative disorder amyotrophic lateral sclerosis (ALS) remains predominantly a clinical diagnosis with significant heterogeneity in rate of disability accumulation. Therapeutic trials rely on survival or change in disability accumulation rate as the primary endpoints. Biomarkers are therefore a research priority (1). Research-based magnetic resonance imaging (MRI) techniques, in particular diffusion tensor imaging (DTI), have demonstrated a consistent involvement of the corticospinal tracts (CSTs) and corpus callosum (CC) in ALS across a range of phenotypes (reviewed in Reference (2)).

Classical ALS is characterized by the presence of both upper motor neuron (UMN) and lower motor neuron (LMN) clinical signs (3). Current diagnostic criteria rely heavily on the presence of UMN signs (4), but these may be difficult to elicit and are minimal or absent in a substantial proportion of ALS cases (5). Those with clinically LMN predominant forms of ALS have a similar clinical progression and postmortem evidence of CST involvement 6, 7, yet may be excluded from therapeutic trials.

In a very small proportion of cases, termed primary lateral sclerosis (PLS), there are only UMN signs clinically and consistently slower overall progression (8). Conversely, the “flail arm” variant of ALS is also typically slower in rate of progression, but predominantly LMN clinically with the main burden of disability characteristically confined to the upper limbs and symmetrical for a period of years (9). In this and all other subtypes of ALS, investigations are aimed at the exclusion of mimic disorders (10), typically involving MRI to consider structural lesion mimics (11). An objective marker of UMN involvement that could be derived from a routine MRI sequence might have value diagnostically in stratification and in assessing the effects of candidate therapeutics.

T2-weighted hyperintensity of the CST was the first documented abnormality in standard clinical MRI sequences in ALS (12), but proved insufficiently sensitive based on only visual inspection. Nonetheless, recent studies have begun to revisit the diagnostic potential of T2-weighted sequences in ALS (13). Our hypothesis was that a quantitative approach to assessing fluid-attenuated inversion recovery (FLAIR) intensity has diagnostic and stratification biomarker potential in ALS. Our aim was therefore to apply this technique to the principal motor tracts of the brain in ALS patients across a range of clinical phenotypes and a group of healthy controls.

## Methods

### Participants

Existing and newly diagnosed ALS patients were recruited from a tertiary ALS referral clinic, diagnosed by experienced neurologists according to standard criteria 4, 14. Two patients with a family history of ALS were found to carry an expansion in the hexanucleotide repeat sequence in *C9orf72*, but all others reported no family history of ALS or frontotemporal dementia (FTD) and were assumed to be sporadic. Similar age and gender healthy controls, typically friends and spouses of patients, were recruited for comparison. Two healthy control participants felt to have deep white matter leukoaraiosis out of proportion to age in the opinion of an experienced neurologist were a priori excluded to avoid confounding white matter intensity quantification.

Ethical approval for all procedures was obtained prior to study (South Oxfordshire Research Ethics Committee 08/H0605/85). Written informed consent was obtained from all participants, and all research was carried out in the UK.

All participants were studied soon after recruitment to the study, and the patients on further occasions at approximately six monthly intervals. All patients underwent clinical examination on the day of attendance. Disability was assessed using the revised Amyotrophic Lateral Sclerosis Functional Rating Scale (ALSFRS-R, scores 0–48 with lower scores reflecting higher disability). Clinical UMN involvement was assessed prior to analysis based on the number of pathological reflexes elicited from 15 body sites: glabella, orbicularis oris, masseter (jaw jerk); plus biceps, triceps, finger, knee, ankle jerks, and Babinski responses bilaterally (15). Disease duration was calculated from symptom onset to scan date in months. A disability progression rate in relation to symptom onset could be calculated at each attendance based upon the equation ((48 − ALSFRS-R)/(disease duration)). A basic measure of executive dysfunction was provided by the Trail Making Test (TMT) using the Trail B minus Trail A score in seconds (16).

### Image Acquisition

MRI was performed using a 3T Siemens Trio scanner (Siemens AG, Erlangen, Germany) with a 12-channel head coil. FLAIR imaging was acquired in a two-dimensional axial orientation (repetition time/echo time 9000/90 ms, flip angle 150°, field of view 220 mm × 220 mm, in-plane resolution 1.1 × 0.9 mm, slice thickness 3 mm, 2 concatenations, 1 average). Whole-brain diffusion-weighted imaging was performed using a spin echo sequence (repetition time/echo time 9300/94 ms, field of view 192 mm, 2 mm isotropic resolution, b value 1000 s/mm^2^, 60 isotropically distributed gradients). High-resolution three-dimensional T1-weighted MRI scans were acquired using a magnetization-prepared rapid gradient echo sequence (repetition time/echo time 2040/4.7 ms, flip angle 8°, field of view 192 mm, 1 mm isotropic resolution).

### Image Analysis and Statistics

MRI data were analyzed using the FMRIB Software Library (FSL) (17). FLAIR signal intensity for each patient was extracted from two regions of interest (ROIs), the CST and CC. To extract the FLAIR signal intensity in each ROI for every subject, the FLAIR images were transformed into a standard space. The FLAIR image was first rigidly registered to the T1-weighted image of the same subject (using the ‘FLIRT’ tool) and then transformed to standard space using a nonlinear registration of the T1-weighted image to the 1 mm Montreal Neurological Institute (MNI) template (with the ‘FNIRT’ tool (17)). Each FLAIR image was inspected for artifact or anatomical abnormality prior to processing. To minimize the impact of scan-scan variation in overall FLAIR intensity, every value was normalized using the mean intensity of all nonzero voxels within the brain-extracted scan volume, excluding the CST and CC where significant changes were expected to be present.

### Creation of CST ROI

The CST ROI was defined using a common template derived using tractography of the CST in the control subjects (as the CST degeneration associated with ALS may reduce the efficacy of tractography in patients). Probabilistic tractography using DTI was employed to generate a ROI mask covering the CST that was representative of the control population. In each subject, tractography was seeded from the left or right motor cortex with an endpoint (or “waypoint mask”) of the pons. Masks were binarized, then transformed from diffusion space to standard space using transformation matrices derived from the registration of a non–diffusion-weighted volume of each subject's DTI data to 1 mm MNI standard space (using first a linear registration to their T1-weighted image, and then a nonlinear registration to standard space). The transformed masks were then thresholded at 0.5 to avoid the volume increase caused by trilinear interpolation, binarized again, and combined. Any voxel found to represent the CST within three or more individual subject volumes was retained. The CC was defined using the Johns Hopkins University white matter atlas. The standard template for the CST was not a good fit for our patient population, hence the creation of a bespoke mask. Manual cleaning of the mask was also performed to remove corticocerebellar and corticocortical fibers (Supplementary Fig S1).

### Quantitative FLAIR Analysis

Mean FLAIR within ROIs was compared between patient and control groups using univariate analysis of variance (ANOVA) with Dunnett's T3 analysis, assuming nonequal variances and controlling for subject age through the weighted least squares method. Kruskal-Wallis testing was performed for multiple comparisons between phenotypes and FLAIR signal across a range of CST and CC ROIs.

### Clinical Measures

Linear regression of the mean of the FLAIR within each ROI was performed vs disease duration, disease progression, ALSFRS-R, and TMT B − A score. Univariate ANOVA, controlled for age (by weighted least squares), was used to investigate correlations between ROIs and UMN score groups. Similarly, FLAIR was assessed for differences in their values between left and right ROIs that correlated with site of symptom onset using the Mann-Whitney U test.

### Classification Analysis

All statistical analyses were performed in SPSS (version 20 IBM Corp. Armonk NY, USA) and controlled for age. A stepwise “discriminant analysis” approach was used, which is a semiautomated statistical tool that aims to determine a simplified classification equation using the variables that best describe the data. If another variable is highly correlated with these, it is eliminated from the equation as there is no added classification benefit from retaining it. The ability of baseline clinical variables to correctly classify patients as “classical” ALS, “flail arm” ALS, and PLS was tested with and without the addition of mean FLAIR intensity data within anatomical locations. Wilks's lambda method was used with inclusion or exclusion by probability of F (entry 0.05, removal 0.1), with no prior assumptions made about group membership and separate groups applied as a covariance matrix.

### Longitudinal Study

Patients were included in the longitudinal analysis if they underwent two or more scans (i.e., initial scan and at least one follow-up). For each patient, the rate of change of FLAIR measures over time within each ROI was calculated. This was derived from the gradient of the linear regression line of best fit of each MRI modality vs disease duration at time of each scan. All scans for each patient were included in the analysis, regardless of the number performed and the period of study inclusion. This method of analysis thereby accounted for the variable timing and number of scans performed on each patient and the different durations of patient inclusion in the study. Gradients for changes in ALSFRS-R and TMT B − A were calculated in the same manner. Linear regression analysis was then performed to look for correlation between the rate of progression of MRI indices of disease and clinical markers.

## Results

A total of 49 patients and 21 controls were available for analysis. Twenty-one patients had at least one follow-up scan. Participant details including patient subgroups are shown in Table 1. The control, cross-sectional, and longitudinal demographics were comparable. The patient cohort was older (60.9 ± 11.1 vs 51.1 ± 12.7 years, *P* = 0.002), and a correction for this was made in the analysis.

**Table 1:** (a) Participant Characteristics (Cross-Sectional Group). (b) Participant Characteristics (Longitudinal Group)

(a) Participant Characteristics (Cross-Sectional Group)					
	Controls (*n* = 21)		Cross-Sectional Patients (*n* = 49)		
	Mean ± SD	Range	Mean ± SD	Range	*P*†
Age (years)*	51.1 ± 12.7	28–72	60.9 ± 11.1	31–83	0.002
Classical ALS	—	—	59.3 ± 11.0	31–77	0.021
“Flail arm” ALS	—	—	61.7 ± 12.5	41–83	0.042
PLS	—	—	68.8 ± 6.2	62–76	0.003
Disease duration (months)*	—	—	59 ± 66.4	5–366	—
UMN score*	—	—	9.5 ± 4.7	0–25	—
ALSFRS-R*	—	—	33.3 ± 5.8	18–44	—
TMT B − A*	—	—	36.8 ± 22	0–86	—

	*n*	%	*n*	%	*P*
Male	11	52.4%	31	63.3%	0.433‡
Classical ALS	—	—	33	67.4%	—
“Flail arm” ALS	—	—	10	20.4%	—
PLS	—	—	6	12.2%	—

			Disease Duration (Months ± SD)		
Classical ALS			35.1	±29.2	
“Flail arm” ALS			64.7	±34.6	
PLS			180.5	±114.9	

(b) Participant Characteristics (Longitudinal Group)								
	Controls (*n* = 21)		Single-Scan Patients (*n* = 28)			Longitudinal Patients (*n* = 21)		
	Mean ± SD	Range	Mean ± SD	Range	*P*†	Mean ± SD	Range	*P*§
Age (years)*	51.1 ± 12.7	28–72	60.9 ± 10.6	31–77	0.143	61.6 ± 12.2	39–83	0.954
Disease duration (months)*	—	—	37.3 ± 39.1	5–190	—	91.5 ± 84	21–366	0.007
UMN score*	—	—	10.0 ± 5.0	2–25	—	8.7 ± 4.7	0–15	0.494
ALSFRS-R*	—	—	33.2 ± 7.2	18–44	—	33.3 ± 3.4	26–37	0.906
TMT B − A*	—	—	39.5 ± 22.5	8–81	—	36.4 ± 19.9	10–86	0.472
Number of scans	—	—	—	—	—	3.6 ± 1.1	2–5	—

	*n*	%	*n*	%	*P*‡	*n*	%	*P*¶
Male	11	52.4%	19	67.7%	0.376	11	52.4%	0.553
Classical ALS	—	—	24	85.7%	—	9	42.9%	0.002
“Flail arm” ALS	—	—	3	10.7%	—	7	33.3%	0.076
PLS	—	—	1	3.6%	—	5	23.8%	0.072

			Disease Duration (Months ± SD)			Disease Duration		*P*‖
Classical ALS			29.8	± 25.0		49.4	±36.0	0.222
“Flail arm” ALS			46.8	± 30.5		72.3	±35.4	0.665
PLS			189.9	—		178.6	128.4	0.980

Longitudinal Patient Clinical Progression			
	*n*	Mean ± SD	Range
Rate of change of ALSFRS**	20	−0.379 ±9 0.25	−1.07 to −0.11
Rate of change of TMT B − A**	14	0.31 ± 1.7	−4.09 to 1.95
Rate of change of CST FLAIR††	21	1.47 ± 2.78	—
Rate of change of CC FLAIR††	21	0.58 ± 3.08	—

ALS, amyotrophic lateral sclerosis; ALSFRS-R, revised Amyotrophic Lateral Sclerosis Functional Rating Scale; CC, corpus callosum; CST, corticospinal tract; FLAIR, fluid-attenuated inversion recovery; PLS, primary lateral sclerosis; SD, standard deviation; TMT, Trail Making Test; UMN, upper motor neuron.

ALS vs PLS: *P* = 0.049.

ALS vs Flail arm: *P* = 0.589.

Flail arm vs PLS: *P* = 0.149.

* Values at first scan.

† Comparison to control population, independent samples *t* test.

‡ Comparison to control population, Fisher's exact test.

§ Comparison to cross-sectional patient population that underwent a single scan (*n* = 28), independent samples *t* test.

¶ Comparison to cross-sectional patient population that underwent a single scan (*n* = 28), Fisher's exact test.

‖ Comparison between same disease subgroup to cross-sectional patient population that underwent a single scan (*n* = 28), independent samples *t* test.

** Individual patient rate of change of clinical score per month of disease.

†† Population mean rate of change of magnetic resonance imaging intensity per month of disease × 10^−3^.

### Quantitative FLAIR Analysis

**Figure 1:**
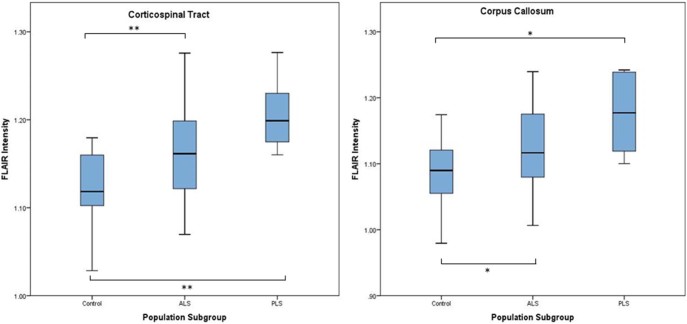
Quantitative corticospinal tract and corpus callosum FLAIR intensity in controls, ALS (including flail arm), and PLS. *P ≤ 0.05; **P ≤ 0.01. ALS, amyotrophic lateral sclerosis; FLAIR, fluid-attenuated inversion recovery; PLS, primary lateral sclerosis.

Age-corrected mean FLAIR intensity within all patients was increased relative to healthy controls in both the CST (1.167 vs 1.121, *P* = 0.001) and the CC (1.132 vs 1.085, *P* = 0.003). There was no significant difference between left and right CST for any group. Subgroup analysis revealed that classical ALS, flail arm ALS, and PLS patients all had higher mean FLAIR intensities than controls in both ROIs, although this did not reach significance for the flail arm group (data not shown). PLS patients had the highest intensity difference in all ROIs (Fig 1). The corona radiata and the body of the CC provided the best subgroup discrimination (Table 2) although Kruskal-Wallis multiple comparisons (classical ALS vs flail arm ALS vs PLS) highlighted only the cerebral peduncles in this respect (*P* = 0.023).

**Table 2:** Quantitative FLAIR in Subregions of the CST and CC

	Control	All ALS Patients		Classical ALS		Flail Arm		PLS	
	Intensity	Intensity	P*	Intensity	P*	Intensity	P*	Intensity	P*
Whole CST	1.121	1.167	0.001	1.167	0.003	1.144	0.114	1.207	<0.001
Corona radiata	1.154	1.210	<0.001	1.208	0.001	1.188	0.038	1.258	<0.001
Internal capsule	1.066	1.091	0.081	1.094	0.084	1.069	0.625	1.114	0.022
Cerebral peduncle	0.984	1.010	0.099	1.017	0.046	0.972	0.462	1.034	0.032
Whole CC	1.085	1.132	0.003	1.127	0.007	1.122	0.070	1.176	0.001
Genu of CC	1.114	1.168	0.009	1.157	0.050	1.176	0.019	1.217	0.001
Body of CC	1.149	1.202	0.001	1.199	0.002	1.191	0.060	1.237	0.001
Splenium of CC	0.996	1.032	0.025	1.029	0.033	1.011	0.386	1.081	0.004

	Control	All ALS Patients		Classical ALS		Flail Arm		PLS	
	Intensity	Intensity	*P*†	Intensity	*P*†	Intensity	*P*†	Intensity	*P*†
Whole CST	1.12								
0–30 months	—	1.16	0.013	1.16	0.009	1.11	0.579	—	—
30–100 months	—	1.17	0.001	1.16	0.011	1.14	0.277	1.25	<0.001
>100 months	—	1.18	<0.001	1.14	0.460	1.19	0.002	1.20	<0.001
Whole CC	1.08								
0–30 months	—	1.12	0.020	1.12	0.009	1.06	0.041	—	—
30–100 months	—	1.12	0.020	1.11	0.133	1.11	0.283	1.18	<0.001
>100 months	–	1.15	<0.001	1.10	0.672	1.16	0.001	1.18	<0.001

ALS, amyotrophic lateral sclerosis; CC, corpus callosum; CST, corticospinal tract; PLS, primary lateral sclerosis.

* Intensity compared to control, univariate analysis, normalized for age.

† Independent samples *t* test, not assuming equal variances, two-tailed significance; compared to control.

### Clinical Measures

**Figure 2:**
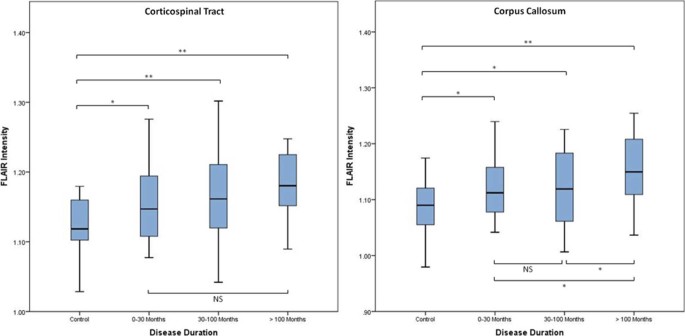
FLAIR intensity in controls vs patients (including all ALS, flail arm, and PLS patients) stratified according to disease duration. All comparisons by independent samples *t* testing, two tailed. *P ≤ 0.05; **P ≤ 0.01; ALS, amyotrophic lateral sclerosis; FLAIR, fluid-attenuated inversion recovery; NS, nonsignificant; PLS, primary lateral sclerosis.

No significant correlation was found for FLAIR intensity in relation to UMN score or the side of first symptoms. Significant increases in FLAIR intensity in the CC were seen with longer disease duration within the amalgamated patient group (Fig 2).

### Classification Analysis

Stepwise “discriminant analysis” showed that rate of change of ALSFRS-R and baseline UMN score were the most useful clinical variables to classify the data into subgroups. This showed a 55% accuracy. Adding in the mean FLAIR intensity data in the anatomical locations shown in Table 2 as variables into the stepwise analysis showed that the FLAIR intensity within the cerebral peduncles and in the genu of the CC improved the accuracy to 82%, with sensitivity and specificity of at least 70% for all patient subgroups.

### Longitudinal Analysis

**Figure 3:**
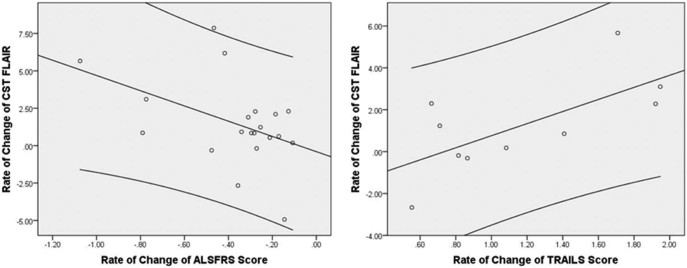
Regression analysis of rate of change of CST FLAIR intensity over time vs rate of decline of ALSFRS (R^2^ = 0.198, *P* = 0.05) and vs rate of change in trail making test, B − A score (R^2^ = 0.458, *P* = 0.03). x axis: rate of change of clinical score per month of disease; y axis: rate of change of MRI intensity per month of disease × 10^−3^. Left panel: A single outlier with a very high rate of ALSFRS-R decline >2 points per month was excluded. Right panel: Only 10 patients were able to complete the TMT on their second visit, hence the smaller dataset. ALSFRS-R, revised Amyotrophic Lateral Sclerosis Functional Rating Scale; CST, corticospinal tract; FLAIR, fluid-attenuated inversion recovery; MRI, magnetic resonance imaging; TMT, Trail Making Test.

FLAIR intensity increased with time. Within the CST, the rate of change of FLAIR intensity over time correlated weakly with rate of change of ALSFRS-R (R^2^ = 0.198, *P* = 0.05), but more strongly with rate of change of TMT B − A score (R^2^ = 0.458, *P* = 0.03) for the patient population as a whole (Fig 3). No correlation was found for any measure within the CC.

## Discussion

This novel quantitative analysis demonstrated that FLAIR intensity is increased in the CST and the CC in ALS patients compared to healthy controls. FLAIR intensity within the cerebral peduncles and genu of the CC most improved the accuracy of the diagnostic discriminant analysis. The small subgroup of PLS patients demonstrated some of the highest FLAIR intensities, but there was no simple relationship with burden of UMN signs. The rate of change of CST intensity in the patients correlated with the rate of change of both physical disability and executive dysfunction.

The routine clinical use of T2-weighted MRI hyperintensity has been constrained by a qualitative sensitivity and specificity of less than 40% and 70%, respectively (18).

Studies taking a more analytical approach to CST intensity have reported more positive outcomes, and noted higher values in those with PLS. The best means of detecting hyperintensity is a matter of debate, with some groups proposing PD-weighted 19, 20, 21, 22, T2-weighted (23), or FLAIR imaging (24) as the most sensitive.

The exact nature of the intensity changes in T2-weighted MRI imaging seen in ALS has yet to be fully understood, although the similarly selective involvement of the CST (and its frontal lobe projections) in hepatic failure has been linked to a potentially common mechanism of oxidative stress (25). Studies in the transgenic SOD1 mouse model of ALS suggest that T2-weighted changes are most closely linked to vacuolation, astrocyte, and microglial activation (26). Changes in DTI measures are thought to reflect axonal breakdown increasing overall diffusivity combined with reactive changes in glial cells and the extracellular matrix as part of the disease pathophysiology (27). The high degree of spatial correlation between FLAIR and published DTI changes in ALS makes it likely that both modalities are caused by the same underlying degenerative pathophysiology. A combined study of T2-weighted hyperintensity and DTI measures supported this view (28). Postmortem MRI combined with histopathology may help shed further light on these issues (29).

Numerous studies have outlined the correlation between DTI changes and ALS phenotype, including clinical UMN involvement (summarized in Reference (30)). Changes in both CC and CST have been linked to extramotor as well as motor deficits in ALS 31, 32. These correlations have marked DTI as a leading source for diagnostic or stratification biomarkers in ALS (33), though meta-analysis reported sensitivity and specificity of less than 70% (34), and DTI's sensitivity to longitudinal change in established cases of ALS may be limited (15). The lack of relationship between UMN scores and FLAIR signal has been noted by others (24), and there is a wider recognition of the limits of neuroimaging and clinical correlations in ALS (35). We have previously found the posterior limb of the internal capsule to have value in stratifying ALS patients using DTI (36), whereas the body of the CC and the corona radiata showed the greatest progressive changes in FLAIR intensity, with the cerebral peduncles providing diagnostic discrimination. The finding of highest FLAIR intensities in PLS patients, particularly in the CC, is of note building on other neuroimaging research findings in this subgroup 32, 37, 38, 39, 40, 41. There is a need for earlier diagnosis in PLS patients to improve the feasibility of earlier therapeutic intervention as well as care planning.

It is accepted that ALS has clinical and pathological overlap with FTD. Loss of executive functions is well described and frequently linked to DTI changes 31, 42. Our study predated the development of focused neuropsychological assessment tools in ALS, such as the Edinburgh Cognitive and Behavioural ALS Screen. Nonetheless, the finding that rate of change of FLAIR is related to rate of decline of a well-established measure of executive dysfunction as well as disability, is in keeping with the observation of worse prognosis in those with executive impairments (43).

We acknowledge the limitations of this study. There were relatively small numbers of participants in the flail arm and PLS subgroups, as well as the longitudinal cohort. There was a mean age difference approaching 10 years between the patient and control groups, although this was a correction factor in the analysis. A more comprehensive cognitive (and behavioral) assessment, and direct comparison with other MRI markers of white matter pathology, for example, Apparent Diffusion Coefficient (ADC) and DTI, would be desirable in future studies.

## Conclusions

DTI remains the leading white matter analysis for the exploratory deep phenotyping of established ALS cases, but FLAIR offers a routine clinical sequence frequently used during the diagnostic work-up of those suspected to have ALS that might offer some added value. An important initiative will be to study such routinely acquired clinical images to see if quantitative analysis can be applied meaningfully (22). Particular challenges will be the development of standardized external templates for coregistration, and automation of intensity standardization across the whole brain. Validation will necessitate applying the parameters derived from this classification analysis to an independent cohort of patients to assess diagnostic accuracy. Comparison with subjects with other neurological diseases, rather than healthy controls, is another key aspect of the aspiration of clinical translation of these findings.

## Appendix Supplementary Data

Figure S1 Image showing corticospinal tract and corpus callosum regions of interest. Figure S1
