# Supplementary figures and images for: Quantitative FLAIR MRI in Amyotrophic Lateral Sclerosis
Source: Acad Radiol. 2017 Oct;24(10):1187–94. doi: 10.1016/j.acra.2017.04.008 (PMC5605225; doi:10.1016/j.acra.2017.04.008)

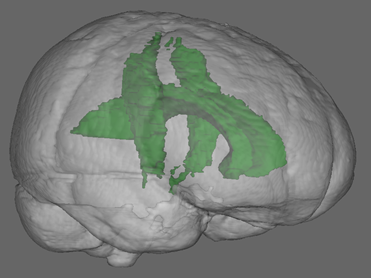

Supplement: Figure S1 — Image showing corticospinal tract and corpus callosum regions of interest. [file mmc1.zip › mmc1.tif]
